# Oncological and surgical outcome after treatment of pelvic sarcomas

**DOI:** 10.1371/journal.pone.0172203

**Published:** 2017-02-15

**Authors:** Stephan E. Puchner, Philipp T. Funovics, Christoph Böhler, Alexandra Kaider, Christoph Stihsen, Gerhard M. Hobusch, Joannis Panotopoulos, Reinhard Windhager

**Affiliations:** 1 Department of Orthopedics, Medical University of Vienna Waehringer Guertel 18–20 Vienna. Austria; 2 Center for Medical Statistics, Informatics, and Intelligent Systems—Section for Clinical Biometrics, Medical University of Vienna Waehringer Guertel 18–20 Vienna. Austria; Universite de Nantes, FRANCE

## Abstract

**Background and objectives:**

Treatment of pelvic tumors remains challenging due to complex anatomy, poor oncological outcome and high complication rates. We sought to investigate the long-term oncological and surgical outcome of these patients.

**Methods:**

Between 1980 and 2012, 147 patients underwent surgical treatment for pelvic sarcoma. Histological diagnosis was Chondrosarcoma in 54, Ewing’s Sarcoma/PNET in 37, Osterosarcoma in 32 and others in 24 patients. Statistical analysis for the evaluation of oncological and surgical outcome was performed by applying Cox proportional hazards regression and Fine-Gray regression models for competing risk (CR) endpoints.

**Results:**

The estimated overall survival (OS) to death was 80%, 45% and 37% at 1, 5 and 10 years, respectively. Univariate analyses revealed a statistically significant unadjusted influence of age age (p = 0.038; HR = 1.01), margin (p = 0.043; HR = 0.51) and grade (p = 0.001; HR = 2.27) on OS. Considering the multivariable model, grade (p = 0.005; HR = 3.04) and tumor volume (p = 0.014; HR = 1.18) presented themselves as independent prognostic factors on OS. CR analysis showed a cumulative incidence for major complication of 31% at 5 years. Endoprosthetic reconstruction had a higher risk for experiencing a major complication (p<0.0001) and infection (p = 0.001).

**Conclusions:**

Pelvic resections are still associated with a high incidence of complications. Patients with pelvic reconstruction and high volume tumors are especially at risk. Consequently, a cautious decision-making process is necessary when indicating pelvic reconstruction, although a restrictive approach to pelvic reconstruction is not necessarily reasonable when the other option is major amputation.

## Introduction

Ten to 15% of all primary bone tumors are located in the pelvic bone of which chondrosarcoma in adults, Ewing’s sarcoma in children, and osteosarcoma in adolescents represent the most common histological subtypes [1–7]. Even today, treatment of pelvic sarcomas remains one of the most predominant challenges for orthopedic oncologists due to the proximity of visceral organs and neurovascular structures [8, 9]. Due to the absence of reliable adjuvant treatment options hindquarter amputation used to be the choice of treatment for malignant pelvic tumors until the early 1980s [10]. With further preoperative imaging and modern multimodality treatment, limb salvage surgery has become a feasible treatment option in these patients [1, 7, 8, 10–18]. Various reconstruction methods have been described, yet not every defect of pelvic resection necessarily requires reconstruction. Ideally, pelvic resection would achieve wide tumor margins followed by anatomic reconstruction whenever possible, leading to a restoration of pre-operative function and quality of life. However, selected tumors involving neurovascular structures still require external hemipelvectomy [9, 10, 19].

In this context, surgery of pelvic bone tumors alone has already been reported to be associated with high revision rates due to complications, while additional reconstruction techniques may even further increase the number of complications. These include intraoperative complications due to neurovascular and visceral compromise, a variety of mechanical and structural failures after endoprosthetic or biological reconstruction, large soft tissue defects leading to the necessity of wound revision or surgical tissue transfer, and infection, which has been reported as the most frequent postoperative complication [1–3, 5, 13–33]. Finally, many authors have stated inferior oncological survival rates for patients with pelvic tumors compared to patients with the same entities in the appendicular skeleton, due to the inability to achieve negative margins, the prolonged diagnostic time spans, larger tumor size or even potentially more aggressive biological tumor behavior [1, 5, 7, 9, 12, 15, 17, 23, 31].

With a relatively low number of large-scale investigations in this area, we performed a retrospective single-center cohort study of a large consecutive series of patients with primary malignant tumors of the pelvis. With this study we sought to investigate: (1) the oncological long-term outcome of patients after resection of a pelvic sarcoma; (2) the surgical outcome and, especially, what type of complications occurred in patients following these procedures; (3) the estimated risks of complications when using a competing risk (CR) model.

## Materials and methods

In a retrospective cohort study, we reviewed 147 consecutive cases with surgical treatment of a sarcoma of the pelvis between 1980 and 2012. All medical records and data were reviewed. Approval of the institutional review board was obtained prior to this investigation (Ethical Review Board—Medical University of Vienna—EK Nr: 767/2008). All patient information and records were anonymized and de-identified prior to analysis.

The records included 68 males (46%) and 79 females (54%) with an average age of 38±20 years (median: 36 years; range: 2–80 years) at time of surgery (Table 1). The median follow-up was 83 months (interquartile range: 20.5–134.0 months). Twelve patients (8%) had distant metastases at the time of diagnosis.

**Table 1:** Baseline characteristics and operative data.

Variable	
**Patients (number; %)**	**147**
**Sex**	
Males	68 (46%)
Females	79 (54%)
**Histology**	
Chondrosarcoma	54 (37%)
Ewing’s sarcoma/PNET	37 (25%)
Osteosarcoma	32 (22%)
Leiomyosarcoma	4 (3%)
Sarcoma-Not other specified	4 (3%)
Hemangiopericytoma	3 (2%)
Others	13 (9%)
**Grading**	** **
G3	101 (69%)
G2	38 (26%)
G1	8 (5%)
**Age at time of surgery (Years; SD)**	38±20
**Size (cm**^**3**^**; SD)**	1023±1848
**Location**	
Ileum	110 (75%)
Ischium	9 (6%)
Pubis	28 (19%)
Periacetabular involvement	67 (46%)
**Type of surgery**	
Resection without reconstruction	46 (31%)
Endoprosthetic reconstruction	47 (32%)
Biological reconstruction	21 (14%)
Internal hemipelvectomy and transposition of the hip	14 (10%)
External hemipelvectomy	19 (13%)
**Type of resection**	
Type I	27 (18%)
Type III	14 (10%)
Type I/II	19 (13%)
Type I/IV	10 (7%)
Type I/II/IV	5 (3%)
Type II/III	25 (17%)
Type I/II/III	33 (22%)
Type I/II/III/IV	14 (10%)

Diagnosis was based on conclusive clinical and imaging findings and was always confirmed by biopsy and histological analysis. Treatment decisions were established by a multidisciplinary council, involving surgeons, oncologists, radiologists, radiotherapists and pathologists. Adjuvant chemotherapy combined with radiotherapy was applied in 35 (24%) patients. Chemotherapy as the only adjuvant was applied in 46 patients (31%). Post-operative radiotherapy as the only adjuvant was applied in 9 patients (6%) and none of the patients had received neo-adjuvant radiation. Surgery was always performed according to the principals of musculoskeletal tumor surgery and surgical margins were evaluated according to the method of Enneking [34]. The type of surgical resection was classified according to the system proposed by Enneking and Dunham (type I: the supraacetabular ilium, type II: the periactabular region; type III: the ischium, inferior, and superior pubic rami; type IV: the sacral massa lateralis; or a combination of these types) [8].

Complications were classified as: (1) infection; (2) neurovascular (neurologic, vascular and thromboembolic events); and (3) mechanical complications (for patients undergoing reconstruction).

### Statistical considerations

Statistical analysis of the data focused on the surgical and oncological treatment outcome of resection of pelvic sarcomas. Demographic variables (sex, age and follow-up), therapeutic variables (reconstruction of the resulting defect, chemotherapy, radiotherapy), and pathological variables (grade, tumor volume, resection margins, local recurrence, metastatic disease and death of disease) were examined. The investigated endpoints of the study were death, progression of disease, first major complication of any cause, and revision for infection or neurovascular complication. For the 68 patients who had a reconstruction of the resulting pelvic defect, a subgroup analysis for mechanical complication as endpoint was performed. Descriptive statistics compromised means and frequencies.

The median follow-up time was estimated using the inverse Kaplan-Meier method [35]. The probability of overall survival to death (OS) was estimated using the Kaplan-Meier (KM) method and univariate and multivariable Cox regression models were performed to evaluate the influence of potential prognostic factors on overall survival. With respect to OS, the prognostic factors of age, sex (male / female), histology (Chondrosarcoma, Ewing’s Sarcoma, Osteosarcoma, others), resection margin (negative / positive), reconstruction (yes / no), grading (3 vs. 2) and tumor volume (log2-transformed) were considered in the regression models. Cases with grade 1 were excluded from analysis due to a low incidence of events (n = 8).

The probabilities of experiencing metastatic disease, local recurrence or complications were estimated by cumulative incidences using the competing risk (CR) approach, where death was considered as the competing event. Varying types of complications were not considered as competing events, as patients sustaining a specific type of complication were still regarded at unaltered risk to subsequently sustain a different type of complication. The time interval to a corresponding complication endpoint was defined as the time from the first surgery to the first occurrence of the complication of interest, regardless of the potential occurrence of other complications meanwhile [36]. The Gray test was used to compare differences with respect to the cumulative incidences between groups of patients. Separate univariate and multivariable Fine-Gray regression models for CR endpoints were applied, evaluating the influence of the potential prognostic factors of age, resection margin, reconstruction, grading, and tumor volume on the probability of the first major complication of any cause and on the probability of revision for infection, respectively. The Firth’s bias correction was used to correct the rather low number of events in the multivariable Fine-Gray models [37].

All statistical tests were two-sided. A p-value of <0.05 was considered statistically significant. The SAS software (SAS 9.4, (2002–2012). SAS Institute Inc. Cary. NC. USA) was used for statistical analysis.

## Results

### Oncological outcome

**Fig 1:**
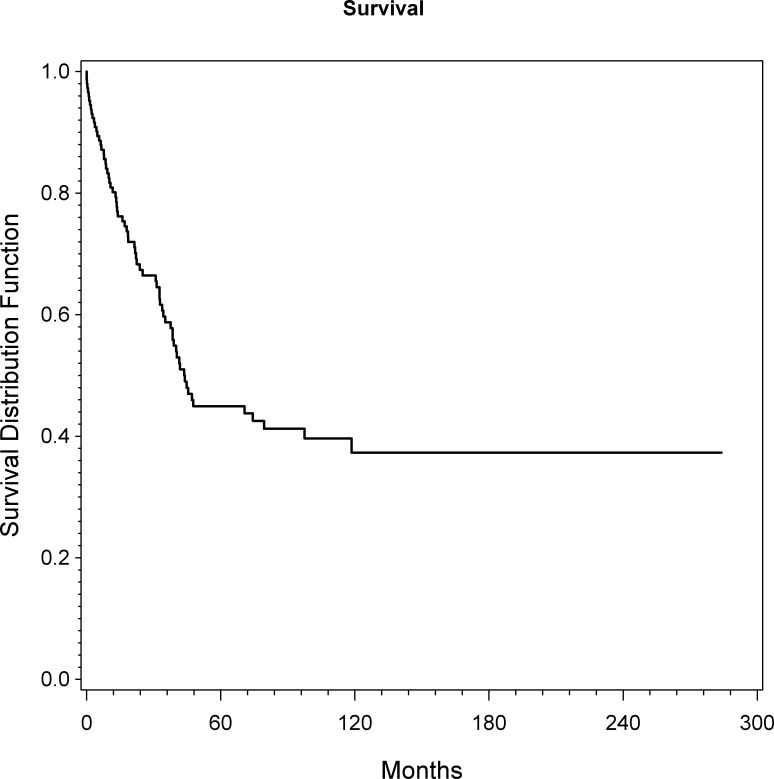
Overall survival of all patients as assessed by KM analysis.

**Fig 2:**
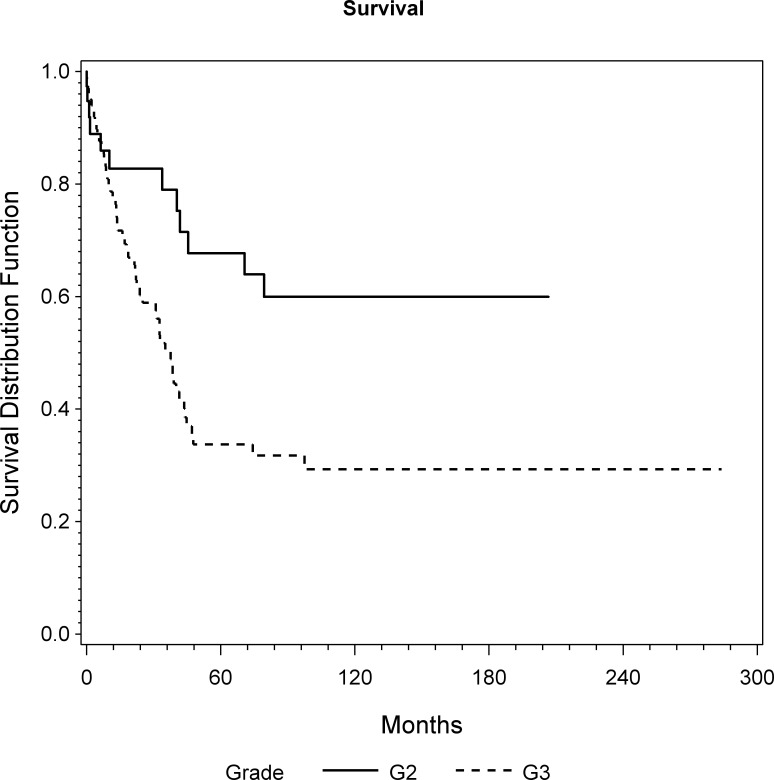
Overall survival of patients with grade 2 and grade 3 as assessed by KM analysis (p = 0.005; HR = 3.04).

The overall 1, 5 and 10 year survival rates were 80%, 45% and 37%, respectively. The median survival was 44 months (Fig 1). At the latest follow-up 70 patients (48%) had died of disease (DOD). Univariate analyses revealed a statistically significant unadjusted influence of age (p = 0.038; HR = 1.01), margin (p = 0.043; HR = 0.51) and grade (p = 0.001; HR = 2.27) on OS (Fig 2). Considering the multivariable model, grade (p = 0.005; HR = 3.04) and tumor volume (p = 0.014; HR = 1.18) are presented as independent prognostic factors on OS (Table 2).

**Table 2:** Univariate and multivariable Cox regression models for overall survival to death.

		univariate			multivariable	
	HR	95%CI	p-value	HR	95%CI	p-value
Overall survival to death						
Age (years)	1,01	1,00–1,03	*0,038	1,01	0,99–1,04	0,220
Sex (male/female)	0,93	0,57–1,50	0,761	0,97	0,59–1,59	0,899
Histology	-	-	0,727	-	-	0,927
Chondrosarcoma	0,78	0,39–1,56	-	0,93	0,45–1,95	-
Ewing’s Sarcoma	0,67	0,32–1,41	-	0,84	0,25–2,87	-
Osteosarcoma	0,90	0,42–1,96	-	1,10	0,42–2,90	-
Margin (neg,/pos,)	0,51	0,27–0,98	**0,043**	0,70	0,34–1,43	0,324
Reconstruction	0,92	0,57–1,49	0,744	1,30	0,73–2,29	0,373
Grade (2 vs. 3)**	2,27	1,21–4,26	**0,001**	3,04	1,41–6,57	*0,005
Tumorvolume (log2-transformed)	1,12	01,00–1,25	0,060	1,18	1,03–1,34	*0,014

*statistically significant

**Grade 1 excluded from analysis due to a low number of events

HR—hazard ratio; CI—confidence interval; neg.—negative; pos.–positive

The estimated probability of experiencing metastatic disease for patients with localized disease at time of surgery (N = 137), according to CR analysis, was 13%, 27% and 32% at 1-, 5- and 10 years, respectively. At the last follow-up, 35 of these patients (26%) had experienced metastases. Patients with chemotherapy (p = 0.002) or grade 3 tumors (p = 0.001) experienced significantly more metastatic disease.

### Type of surgery

Resection without reconstruction was performed in 46 patients (31%); (20 type I resections; 4 type I/II (partial) resections; 1 type I/II/III (partial) resection; 3 type I/IV resections; 6 type II/III resections); 12 type III resections). No reconstruction was commonly indicated in patients where the anatomy of the hip joint could be maintained (tumors in the ischio-pubic region/type I and III resections) or when patients had poor general conditions and a high risk of perioperative morbidity. In five (7%) of these patients, vascular structures were resected and reconstructed. In three patients (2%), an additional synthetic ligament (LARS®) or resorbable mesh (VICRYL Woven Mesh®) was implanted for soft tissue repair. One patient required additional soft tissue coverage by a free muscle flap and one had a split skin graft.

Endoprosthetic reconstruction was indicated in 47 patients (32%); (11 type I/II resections; 8 type I/II/III resections; 5 type I/II/III/IV resections; 5 type I/II/IV resections; 17 type II/III resections; 1 type III resection). Indication for endoprosthetic reconstruction was made in case of acetabular involvement in young patients with high life expectancy. A custom made pelvic prosthesis was implanted in 27 patients (60%). Nine patients (6%) were treated by use of a pedestal cup. In nine patients (6%), a saddle-prosthesis was implanted. In two patients (1%) with a type II/III resection, a roof reinforcement ring was used. Five patients (3%) received an additional synthetic ligament (LARS®) for capsular repair. Four patients (3%) required additional soft tissue coverage by a free muscle flap. Two patients (1%) had a split skin graft. In three patients (2%), vascular structures were resected and reconstructed. An additional iliac crest graft in three patients (2%), a tibia graft in one patient and an allograft in one patient were also implemented.

In 21 patients (14%), biological reconstruction was performed after tumor excision (7 type I resections; 4 type I/II resections; 7 type I/II/III/IV resections; 2 type II/III resections; 1 type III resection). A tibia graft was used in 13 patients (9%), an iliac crest graft in six patients (4%), a fibula graft in one patient and an ischial graft in one patient. In two patients (1%), an additional synthetic ligament (LARS®) or resorbable mesh (VICRYL Woven Mesh®) was implanted for soft tissue repair. Two patients required additional soft tissue coverage by a free muscle flap. Table 1 details baseline characteristics of all patients.

In 14 patients (10%), an internal hemipelvectomy with transposition of the hip was performed (7 type I/II/III resections; 5 type I/II/III/IV resections; 2 type I/II/III partial /IV resections). Internal hemipelvectomy was defined as a complete unilateral resection of the pelvis with or without parts of the lateral sacrum (Type I/II/III and I/II/III/IV). In 12 patients (8%), an additional synthetic ligament (LARS®) was used for reconstruction of a pseudo-capsule attached to the sacrum. One patient required additional soft tissue coverage by a free muscle flap and one had a split skin graft.

External hemipelvectomy had to be performed in 19 patients (13%); (17 type I/II/III resections; 2 type I/II/III/IV resections). This was indicated when neurovascular structures could not be spared due to the intrapelvic extent of the lesion. Three of these patients (2%) required a free muscle flap to restore soft tissue coverage.

### Surgical outcome and cumulative incidence of complications

Negative surgical margins were found in 131 patients (89%). Secondary amputation had to be performed in 13 patients (9%) due to uncontrollable infection in five patients, local recurrence in four patients, thrombosis in two patients, and metastasis in two patients. Consequently, at the last follow-up the overall limb-sparing rate was 78%.

Local recurrence occurred in 17 patients and was only observed in patients with limb sparing surgery versus patients with ablative surgery. The estimated probability of experiencing local recurrence according to CR analysis was 5%, 14% and 14% at one 1-, 5- and 10 years, respectively.

**Fig 3:**
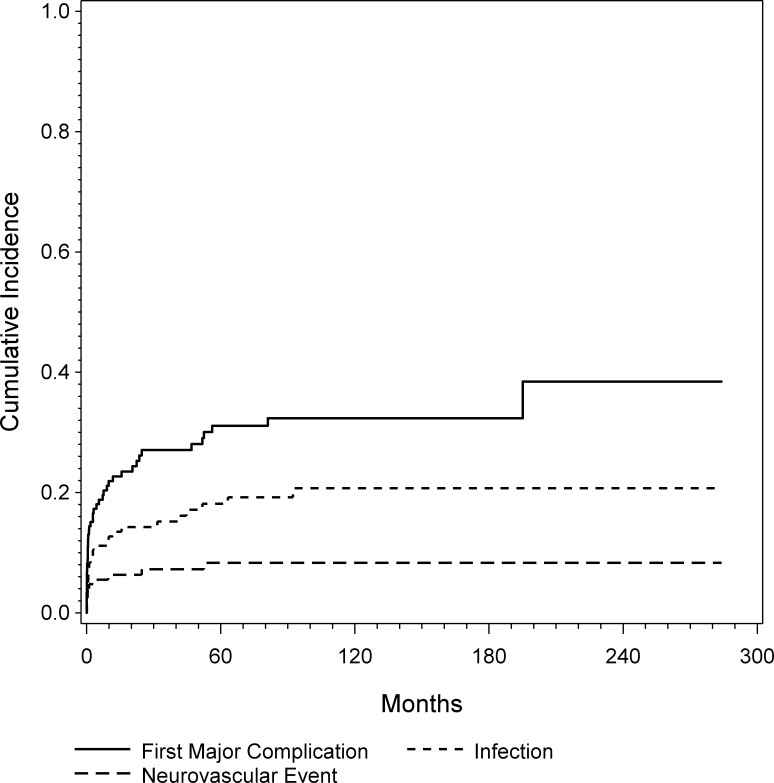
Cumulative incidence of first major complication, infection and neurovascular complication was assessed by CR analysis.

**Fig 4:**
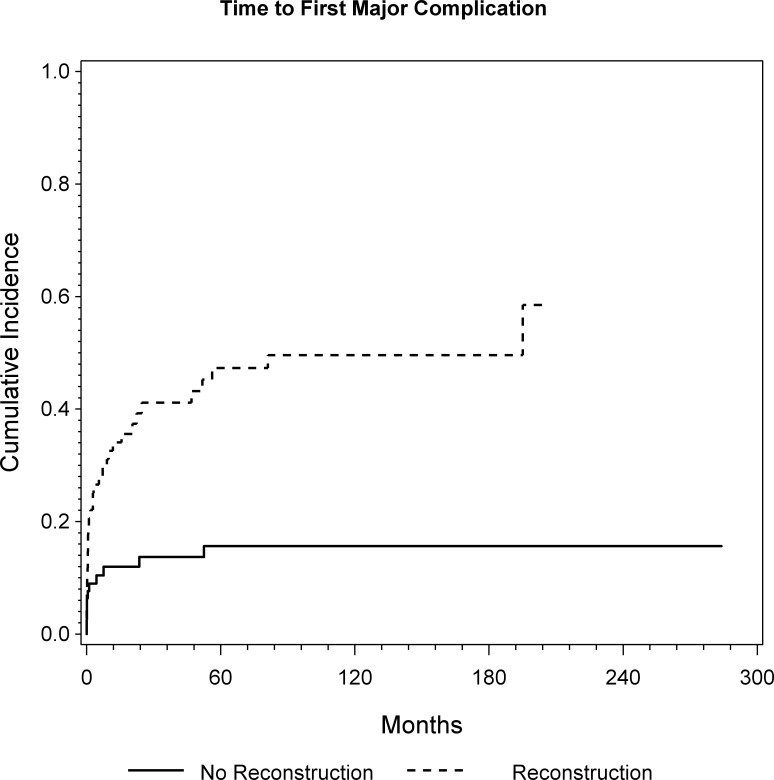
Cumulative incidence of first major complication for patients with and without endoprosthetic reconstruction was assessed by CR analysis (p<0.0001; HR = 4.93).

At the latest follow-up, 43 patients experienced at least one major complication. In 20 patients the complication occurred within one month, in 26 patients within 6 month and 32 patients within one year. CR analysis showed a cumulative probability for a major complication of 23%, 31% and 32% at 1, 5, and 10 years, respectively (Fig 3). The 47 patients who underwent endoprosthetic reconstruction had a significantly higher risk for experiencing a major complication (p<0.0001; HR = 4.93) as shown by univariate and multivariable regression analyses (Fig 4 and Table 3).

**Table 3:** Univariate and multivariable competing risk Fine-Gray regression models for 1st major complication.

		univariate			multivariale	
	HR	95%CI	p-value	HR	95%CI	p-value
1st Major Complication						
Age (years)	1,00	0,98–1,01	0,641	0,99	0,98–1,02	0,937
Margin (neg,/pos,)	1,33	0,51–4,88	0,597	0,93	0,34–3,46	0,892
Surgery						
Endoprosthetic Recon.^+^	4,40	2,19–9,49	*<0,0001	4,93	2,20–9,83	*<0,0001
Biliogocal Recon. ^+^	2,20	0,78–5,74		2,17	0,73–6,05	
Tumorvolume (log2-transformed)	1,02	0,88–1,18	0,756	1,06	0,91–1,24	0,483
Grade (2 vs. 3)**	0,93	0,49–1,93	0,844	0,90	0,44–1,95	0,783

^+^ vs. no reconstruction

*statistically significant

**Grade 1 excluded from analysis due to a low number of events

HR—hazard ratio; CI—confidence interval; neg.—negative; pos.–positive.

**Fig 5:**
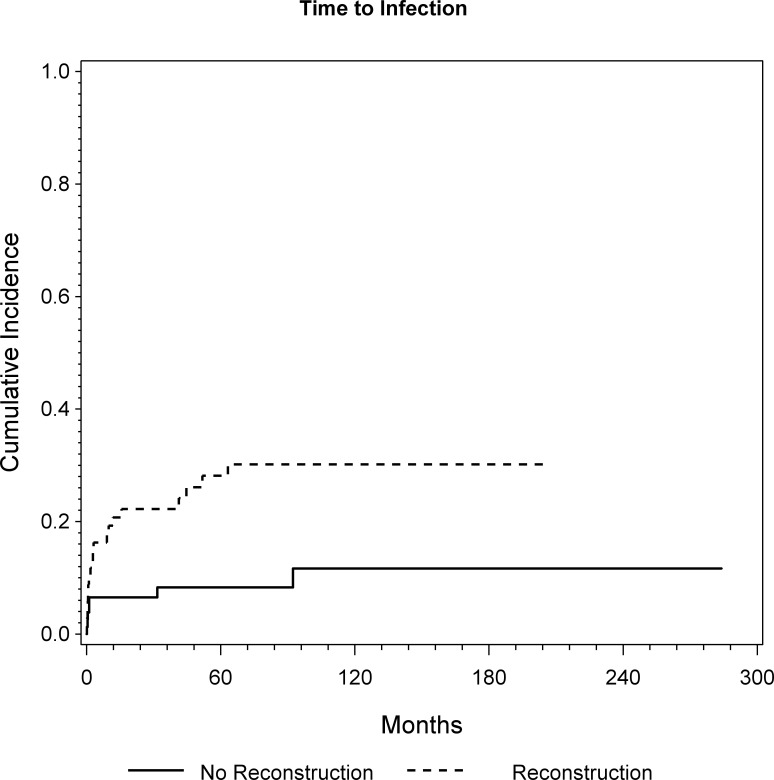
Cumulative incidence of infection for patients with and without endoprosthetic reconstruction was assessed by CR analysis (p = 0.0017; HR = 4.11).

Overall, 26 patients experienced an infection. In 8 patients the complication occurred within one month, in 13 patients within 6 month and 15 patients within one year. Five patients had late infections after more than three years. All five were prior treated with endoprosthetic reconstruction. CR analysis showed a cumulative incidence of infection of 13%, 18% and 21% at 1, 5, and 10 years, respectively (Fig 3). Again, the 47 patients who underwent endoprosthtic reconstruction had a statistically significantly higher risk for experiencing an infection (p = 0.001; HR = 4.11) (Fig 5). Additionally, patients with high volume tumors tended to have a significantly higher risk for experiencing postoperative infection (p = 0.052, HR = 1.23), according to the multivariable regression model.

Eleven patients experienced a neurovascular complication after an average time. In 5 patients the complication occurred immediately postoperative, in 7 within 6 month and 8 within one year. Two patients suffered from late neurovascular complications. One had a rupture of the femoral artery 4,4 years and another a nerve lesion after 2.1 years after surgery. CR analysis showed a cumulative risk of experiencing a neurovascular complication of 6%, 8% and 8% at 1, 5, and 10 years, respectively (Fig 3).

Out of the 68 patients who underwent reconstruction, 15 patients (22%) suffered from mechanical complications. In 5 patients the complication occurred within one month, in 6 patients within 8 month and 15 patients within one year. Four patients (27%) had late infections after more than 3 years. CR analysis showed a cumulative risk of mechanical failure for patients with reconstruction of 12%, 22% and 24% at 1, 5, and 10 years, respectively.

Detailed information regarding complications and surgical procedures are given in Table 4.

**Table 4:** Complications according to the type of surgery.

Complications	Endoprosthetic reconstruction	Biological reconstruction	No reconstruction	Internal hemipelvectomy	External hemipelvectomy
	(N = 47)	(N = 21)	(N = 46)	(N = 14)	(N = 19)
**Major**					
Infection	17 (36%); (* 17 Type IV)	2 (10%)	3 (7%)	3 (21%)	1 (5%)
Neurovascular/Visceral	5 (11%)	-	4 (9%)	1 (7%)	1 (5%)
Mechanical/Soft-Tissue failure	11 (23%); (* 4 Type II / 13 Type III)	4 (19%)	-	-	-
**Minor**					
Woundhealingdisorder	17 (36%)	3 (14%)	9 (19%)	3 (21%)	6 (32%)
Seroma/Hematoma	6 (13%)	6 (28%)	7 (15%)	2 (14%)	2 (11%)

*according to Henderson et al. 2011: softtissue failures (Type 1), aseptic loosening (Type 2), structural failures (Type 3), infection (Type 4), and tumor progression (Type 5).

Tumor histology had no significant influence on first complication, infection, neurovascular complication, or mechanical complication.

## Discussion

The treatment of malignant bone tumors of the pelvis remains a challenging procedure. Regardless of emerging adjuvant therapy strategies and improved imaging technology, the complexity of the treatment of these lesions remains to be reflected by a lower survival rate and a greater number of complications compared to the same tumors at other locations [1, 2, 5, 7, 9, 11, 12, 15–17, 19, 20, 22, 23, 25–27, 29–31, 38]. Despite these facts, limb salvage surgery has evolved as a realistic treatment option [1, 7, 9–17, 38]. The decision regarding which treatment is adequate can be difficult in pelvic tumor surgery and should be made using a multidisciplinary approach. Yet, the preservation of function of the hips and bodies integrity are essential for patients, which might shift the decision towards pelvic reconstruction, although a greater number of complications, followed by revision surgery, have been reported for this type of surgery [2, 5, 7, 8, 10, 14, 15, 20–22, 25]. In this context, we sought to evaluate a large single center cohort of surgically treated pelvic sarcomas. Particularly, we evaluated the oncological outcome, the surgical outcome and the risks of complications when applying a CR model.

The OS to death was 45% at five years. This data is in accordance with literature, where five year OS ranges between 40% and 70% for surgically treated patients [1, 7, 9, 12, 15, 17, 23, 31]. While univariate analyses revealed an influence of age (p = 0.02; HR = 1.01) and grade (p = 0.005; HR = 2.30) on OS, the multivariable model showed that grade (p = 0.003; HR = 2.82) and tumor volume (p = 0.011; HR = 1.18) are independent prognostic factors on OS. Bergh et al have also suggested age as a prognostic factor for OS [39]. In accordance to our results Jawad et al found grade and size to be a significant prognostic factors for OS in their series of 1185 pelvic sarcoma cases [40]. Mavrogenis et al also found that grade was the most important univariate and multivariate predictor of OS in their series of chondrosarcomas oft he pelvis [38]. Likewise did Shin et al, who reported grade as the only independent prognostic factor in their cohort of 46 primary bone sarcomas of the pelvis. Although sometimes contrary discussed in literature, like several other studies, our current data clearly demonstrates with perspicuous statistical significance in univariate and multivariate analysis, that grade is the most important prognostic factor for OS [5, 7, 12].

Similar to Wirbel et al, we did not find any difference in terms of survival concerning the chosen surgical procedure [7]. However, hemipelvectomy was selected when adequate surgical margins could not be expected due to involvement of nerves and large amounts of soft tissue. Thereby a selection bias may result when comparing external hemipelvectoy to limb sparing procedures [7, 8, 10].

Complications after pelvic tumor resection are very common [2, 5, 7, 8, 10, 14, 15, 20–22, 25]. In this series, 29% of the patients suffered from at least one major complication. Especially those patients who underwent endoprosthetic reconstruction had a higher risk of experiencing a major complication (p<0.0001) afterwards. The most common major complication was infection and again, the 46 patients who underwent reconstruction had a higher risk of experiencing an infection in the univariate model (p = 0.0017). Infection may be associated with large and extensive approaches resulting in large cavities accompanied by extended soft tissue resection. In accordance with that, patients with high volume tumors tended to have a significantly higher risk of experiencing postoperative infection (p = 0.052). In accordance with our data, Angelini et al reported reconstruction as the only independent significant prognostic factor of infection after pelvic tumor resection. This fact is supported by numerous published studies [7, 15, 16, 30]. The prolonged surgical time, the proximity of the tumor to the rectum and genitourinary tract, together with a large dead space filled with a large amount of foreign material are all arguments for the high risk of infection, particularly after pelvic reconstruction [30]. Earlier, we were able to show that almost 50% of patients died within 15 months after surgery or were alive with disease and as a consequence, resection should be followed by pelvic reconstruction methods that warrant the lowest complication rate and the earliest possible rehabilitation [15]. Consequently, a cautious and restrictive decision-making process is necessary when indicating pelvic reconstruction, which may be tailored to a large number of serious complications and in succession, would impede the patient’s quality of life, considering the probability of a poor life expectancy.

To analyze the outcomes of pelvic tumor surgery and the corresponding impact of reconstruction, we performed CR analysis. The CR approach for estimating the risk of complications results in a more realistic description, since it accounts for the existence of the competing event of death. These lower failure probabilities–compared to the estimates resulting from the conventional Kaplan-Meier approach–may better reflect reality considering the high competing risk of death of disease in oncologic patients with unfavorable tumors [41]. Yet further studies using this statistical analysis are needed to compare these results and draw conclusions, which go beyond a single center experience.

We have to acknowledge several limitations of this study. First, this is a retrospective analysis. Nevertheless, accurately reviewed patient data in a retrospective study delivers useful results to obtain conclusion for management of further treatment. A follow-up of less than two years in some patients will still capture all deaths, but will tend to de-emphasize oncologic failures relative to deaths. Moreover, inclusion of all patients treated in the study period, irrespective of their actual length of follow-up, is essential to obtain unbiased estimates of the complication incidences. Second, the group of tumors is heterogeneous. Some patients were administered adjuvants or radiotherapy. Some had a low life expectancy, while others had a high life expectancy. It must be admitted that the interpretation of outcome may be affected by oncological outcomes and life expectancy, yet with CR analyses this bias will be reduced, especially for complication free survival. The fact that the data is invalid because treatments have changed over time may sound reasonable, but in actuality, chemotherapy and radiation for the diagnoses in question have changed very little and it would be impossible to provide a series sufficient for multivariate analysis, as we have done, without using data from a vast time period, at least from past the 1980s.

While this study certainly has the afore mentioned weakness, we believe that it still represents a large series of primary pelvic resections so far and it is the first one to use competing risk analysis in this area. Since prospective randomized controlled trials are not yet available in this treatment area, and might not be in the future, our results can be useful, even after considering of these limitations.

## Conclusions

Summarizing, OS after surgical treatment for pelvic sarcomas is low while complication rates remain high. Patients with pelvic reconstruction and high volume tumors are especially at risk. Consequently, a cautious decision-making process may be necessary when indicating pelvic reconstruction. Yet, a restrictive approach to pelvic reconstruction is not necessarily reasonable, especially when the other option is major amputation.
